# ABILHAND-KIDS YOUNG CP: A MEASURE OF MANUAL ABILITY IN YOUNG CHILDREN WITH CEREBRAL PALSY AGED 2 TO 7

**DOI:** 10.2340/jrm.v57.42691

**Published:** 2025-08-20

**Authors:** Carlyne ARNOULD, Julie PARADIS, Massimo PENTA, Jean-Louis THONNARD, Yannick BLEYENHEUFT

**Affiliations:** 1 Forme & fonctionnement Humain (FfH) Unit, CeREF-Santé, Haute Ecole Louvain en Hainaut, Montignies-sur-Sambre; 2 Institute of Neuroscience, Université catholique de Louvain, Brussels; 3 Department of Occupational Therapy, Haute Ecole Léonard de Vinci, Brussels; 4 Arsalis SRL, Glabais, Belgium

**Keywords:** activities of daily living, cerebral palsy, child, preschool, hand, upper extremity, patient-reported outcome measures

## Abstract

**Objective:**

Hand dysfunction is one of the main factors contributing to daily activity limitations in children with cerebral palsy (CP). As a latent variable, manual ability is not easily measurable, especially in young children. This study aimed to develop ABILHAND-Kids Young CP, a Rasch-built manual ability measure in young children with CP.

**Design:**

Prospective study/questionnaire development.

**Subjects/Patients:**

107 CP children aged 2 to 7 (59% unilateral CP).

**Methods:**

Responses of children’s parents to an 80-item experimental questionnaire were analyzed using RUMM2020 software to select items presenting the best psychometric qualities.

**Results:**

ABILHAND-Kids Young CP includes 17 items with well-discriminated response categories and defines a valid, unidimensional, and linear scale. It presents high measurement precision (R = 0.94) and is invariant allowing the measurement of young children with CP whatever their age, gender, clinical form, and Manual Ability Classification System (MACS) levels. Its measures are significantly related to age (*r* = 0.22), school education, clinical form, MACS (*r* = –0.63) and Pediatric Evaluation of Disability Inventory (*r* = 0.74) (all *p* < 0.001, except age at *p* = 0.023).

**Conclusion:**

ABILHAND-Kids Young CP is a unidimensional and linear scale specifically developed to measure manual ability in young children with CP. Its psychometric properties show promising potential in monitoring children’s evolution related to neurorehabilitation.

## LAY ABSTRACT

Cerebral palsy, resulting from brain lesions during foetal life or infancy, is mainly characterized by motor disorders. Manual ability, referring to the capacity to use the upper limbs in the achievement of daily activities, is a crucial determinant for children’s independence and constitutes one of the primary parents’ rehabilitation priorities. It deserves to be measured in early childhood where rapid developmental gain happens. ABILHAND-Kids Young CP was developed using a sound methodology ensuring that an objective measurement of manual ability in young children with cerebral palsy aged from 2 to 7 could be obtained. ABILHAND-Kids Young CP includes 17 manual activities for which parents are asked to estimate their child’s ease/difficulty in performing them. Its high measurement precision, clinical relevance, and free web access (https://www.rehab-scales.org) offer new opportunities to assess manual ability in children with cerebral palsy, whatever their age (from 2 to 7), gender, clinical form, and manual ability levels.

Cerebral palsy (CP) is the most common cause of long-term physical disability in childhood (1). Up to 60–80% of children with CP experience more than minor hand dysfunctions (2), which may restrict children’s daily activities and social participation and cause considerable distress to the children, their parents, and caregivers. In CP, most problems prioritized by children’s parents refer to daily activities for all age groups (3, 4). More specifically, self-care, leisure, and play tasks are of particularly high priority for parents of young children (3, 4). So, measuring manual ability in CP, namely “the capacity to manage daily activities requiring the use of the upper limbs, whatever the strategies involved” (5), is essential as it constitutes a crucial determinant in self-care performance (6, 7).

ABILHAND-Kids (8), developed for children aged 6 to 15 years, is a parent-report questionnaire measuring the child’s ease or difficulty in performing manual everyday activities. This tool has been developed through the Rasch measurement model (9), a modern psychometric approach to construct instruments in rehabilitation that has become state-of-the-art because it transforms ordinal scores into interval-level measures and verifies that a scale meets the requirements of an objective measurement. Several systematic reviews have concluded that ABILHAND-Kids is a manual performance measure of choice for children aged 6–15 years with unilateral or bilateral CP as it offers excellent clinical utility and robust psychometric properties (10–13). ABILHAND-Kids is inexpensive, quick to administer, easy to use, valid, reliable, unidimensional, and sensitive to change (8, 14). Moreover, ABILHAND-Kids can be freely downloaded and analyzed for private practice in clinic or research on www.rehab-scales.org.

However, ABILHAND-Kids cannot be used for children with CP under the age of 6. Indeed, the item difficulty hierarchy may vary with age and preschool children have very different occupational interests than school-aged children or adolescents and, thus, undertake different activities (15). Therefore, an instrument intended to measure manual ability in young children should include age-appropriate activities. The measurement of manual ability in young children seems essential as early childhood is a period of particularly rapid developmental gain in hand skills, notably in self-care (16). Furthermore, previous CP studies (17, 18) indicate that early manual performance is an important predictor of future hand functioning among children with unilateral and bilateral CP.

Some manual performance tools were specifically developed for young children with CP (Appendix S1). Although these instruments provide relevant clinical information, they are exclusively focused on the assessment of either children with unilateral or bilateral CP (e.g., Infant and Pediatric Motor Activity Log [IMAL/PMAL], Assisting Hand Assessment [AHA], Caregiver Functional Use Survey family tools, or Hand-Use-at-Home questionnaire), capture the mobility rather than the self-care domain (e.g., IMAL/PMAL and AHA family tools), or require further investigations into their psychometric qualities (e.g., self-care domain of Child Engagement in Daily Life Measure – second version [CEDL2]).

The objective of this study was therefore to develop ABILHAND-Kids Young CP, a Rasch-built measure of manual ability in young children with CP aged from 2 to 7.

## METHODS

### Participants

One hundred and seven children with CP aged from 2 to 7 years (61% of boys; mean (SD): 4.7 (1.5) years old) were recruited in this study from 14 centres dedicated to CP in Belgium (*n* = 2) and France (*n* = 12). The study was authorized by the ethics committee of Saint-Luc – UCLouvain, Belgium (clinical trial number: NCT02667613).

Some 75% of children attended regular kindergarten while 11.5% were in specialized education and 13.5% were not schooled. Most of the children (59%) presented unilateral CP. All levels of the Manual Ability Classification System (MACS) were observed in our sample, although the most severe level was less represented. Demographic and clinical characteristics of the sample are presented in Table I.

**Table I:** Sample description (*n* = 107)

Age, years, mean (SD) [range]	4.7 (1.5) [2-7]
2-3 years	29
4-5 years	45
6-7 years	33
Gender	
Male	65
Female	42
Handedness	
Right	45
Left	48
Unknown	14
School education	
Regular	77
Special	12
Home (not schooled)	14
Unknown	4
Clinical form of CP	
Unilateral CP	58
*Right*	*38*
*Left*	*19*
*Unknown*	*1*
Bilateral CP	40
* Tetraplegia/paresis*	*27*
* Diplegia*	*13*
Unknown	9
MACS	
Level I: most independent hand function	27
Level II	27
Level III	20
Level IV	15
Level V: least independent hand function	5
Unknown (too young)	13

CP: cerebral palsy; MACS: Manual Ability Classification System.

### Questionnaire development

ABILHAND-Kids Young CP was designed to cover the widest range of daily activities performed by young children (i.e., eating, dressing, hygiene and grooming, plays and games, leisure activities, various other activities). A preliminary questionnaire included 105 items containing 21 items from the ABILHAND-Kids questionnaire (8), 16 items of the Pediatric Evaluation of Disability Inventory (PEDI) self-care subscale (19), and 68 items added to extend the range of activities of the questionnaire. Some of the additional activities came from observational works on the age of acquisition of manual activities in healthy children (20). All activities included in the preliminary questionnaire had to predominantly involve the upper extremities and to be relevant for all young children.

To ensure scale content validity, the preliminary questionnaire was submitted to 7 experts on children’s manual ability (6 occupational and 1 physical therapists), who were asked to assess the clarity and relevance of the activities and to suggest additional items not included in the preliminary list. Following experts’ review, 30 irrelevant items were removed (rated as relevant by < 80% of the experts), 4 new items were added, 1 item was split into 2 more accurate items, and 12 too vague items were clarified. The experimental version of ABILHAND-Kids Young CP included 80 items.

### Instrument and procedures

The ABILHAND-Kids Young CP experimental version explored unimanual and bimanual activities completed without technical or human assistance. Thus, the questionnaire was not devised for measuring the performance of children wearing a robotic orthosis as part of their daily functioning. The rationale for this choice was to measure manual ability in an autonomous way, avoiding potential bias between the assessments of children who do not have access to the same material/help. The caregivers (usually the parents) were asked to estimate the child’s ease or difficulty in performing each activity independently, irrespective of the limb(s) actually used to do the activity and whatever the strategy implemented (any compensation is allowed), on a 3-level scale: impossible [0], difficult [1] and easy [2]. Unfamiliar activities that were not performed in the last 3 months were not scored and considered as missing responses (8% of the data). The experimental questionnaire was mostly mailed to the respondents (61%) or completed in a waiting room before a consultation (39%).

### Data analysis

*Rasch model.* Caregivers’ responses to the experimental questionnaire were analyzed using the Rasch model with RUMM 2020 (RUMM Laboratory Pty Ltd, Perth, Western Australia). The Rasch model (9), a probabilistic model increasingly used in the development of measurement tools in healthcare, estimates the ability of each person, the difficulty of each item/activity, and their thresholds (i.e., the ability level for which 2 successive response categories have the same occurrence probability) on a common linear scale. The Rating Scale Rasch model (21) has been used to verify whether the scale meets the requirements of objective measurement and to select the items presenting the best psychometric qualities. It also converts the observed ordinal total scores into linear measures expressed in logits, a measurement unit that is constant and reproducible throughout the measurement scale. To provide a more common and understandable measurement unit, the logit unit has been transformed into a percentage of the full range scale (expressed in logits), where 0% represents the lowest manual ability level and 100% the highest.

Successive analyses were used to select items presenting good psychometric qualities to constitute the final ABILHAND-Kids Young CP scale (Appendix S2). Relevant items presenting the following criteria were retained: to display ordered response categories with similar discrimination to other items (i.e., a similar measurement range of the middle category “difficult”), to present an adequate fit between model predicted and observed responses (i.e., unidimensional items), to show an invariant item difficulty hierarchy across age (≤ 4 years old vs. > 4 years old), gender, CP clinical form (hemiplegia vs. diplegia vs. quadriplegia), and MACS levels (MACS = I-II vs. MACS ≥ III), and to demonstrate local independence.

*Item-patient targeting and internal consistency reliability.* The item-patient targeting has been verified by comparing mean children’s measures with mean item difficulty (conventionally set at 0 logit or 50% of full scale) to verify whether the scale difficulty was well adapted to the sample ability. The percentage of children with minimum (0% of full scale) or maximum score (100% of full scale) lower than 15% was considered as non-substantial floor and ceiling effects (22).

The degree of precision achieved by the scale in our sample (i.e., its internal consistency or reliability) was examined by computing the Rasch person separation index. This index was computed as the ratio between the true and observed measure variances and allows the number of manual ability levels that may be statistically distinguished in the sample to be calculated (9). A person separation index ≥ 0.90 indicates high internal consistency.

*Construct validity.* To validate the item difficulty hierarchy, 10 experts (4 physiotherapists and 6 occupational therapists) were independently asked to classify each selected activity according to the hand involvement it requires: unimanual activities (1) and bimanual activities manageable in several unimanual steps (2A), requiring stabilization with 1 hand and digital activity with the other (2B) or requiring digital activity from both hands (2C). We hypothesized that 2-handed activities should display the highest difficulty levels, while unimanual activities or activities manageable in several unimanual steps should be the easiest. Experts were also asked to indicate, for each hand, the level of precision and strength required to perform the activities and to determine if, at least, 1 hand had a stabilizer role. We hypothesized that tasks requiring precision or strength from both hands should be the most difficult while tasks requiring neither precision nor strength from both hands, nor stabilization from at least 1 hand should be the easiest.

Convergent construct validity was also investigated through the relationships between ABILHAND-Kids Young CP measures and the children’s demographic (age, gender, handedness, school education) and clinical (clinical form of CP, MACS levels) indices. We hypothesized that measures should be significantly related to clinical indices and school education but not to gender and handedness. A significant relationship could also be observed with age, but with less certainty because the degree of children’s disability may hide the age-related changes. Convergent validity was additionally investigated by measuring the degree of association between ABILHAND-Kids Young CP and the self-care section of the PEDI (19), a generic measure of children’s ability in self-care daily activities. This information was only available for a subsample of our participants (*n* = 66). Spearman correlation coefficients were computed for continuous indices, *t*-tests or Mann–Whitney tests for 2 groups of nominal indices, and one-way ANOVA or Kruskal–Wallis one-way ANOVA on Ranks for more than 2 groups of nominal indices. Non-parametric statistics were chosen when normality and/or homoscedasticity were not satisfied. The alpha level of significance was set at 0.05 for all statistical analyses performed with SigmaPlot 14.0 (https://alfasoft.com/software/statistics-and-data-analysis/data-visulization/sigmaplot/).

## RESULTS

### Item selection of ABILHAND-Kids Young CP

All items of the experimental questionnaire had a response rate ≥ 80%, indicating that all activities belong to the daily life of most CP children in our sample. Nine items presented disordered response categories, and 4 items did not share a similar rating scale. These 13 items were therefore removed, and a rating scale model was then applied (by forcing all items to share the same relative threshold locations). Twenty-one items were misfitting with the unidimensional scale formed by the other items and 28 additional items presented differential item functioning (DIF) across age (*n* = 14), gender (*n* = 11), CP clinical form (*n* = 7), and MACS levels (*n* = 6), with several items showing DIF across several children’s characteristics (*n* = 8). DIF items are activities whose difficulty varies according to the investigated children’s attributes. They were removed together with misfitting items. Finally, the item “Putting on a T-shirt” was removed as it presented a high local dependence (*r* = 0.51) with the item “Taking off a T-shirt”, which was retained due to its better psychometric qualities (i.e., fewer missing data and better fitting). Thus, a total of 17 items were retained to create a unidimensional scale for young children with CP regardless of their age (from 2 to 7 years), gender, CP clinical form, and MACS levels.

### Calibration and metric properties of ABILHAND-Kids Young CP

The calibration of the 17-item ABILHAND-Kids Young CP scale is presented in Table II. The item difficulties range from 31.4% to 64% of full scale. The scale covers different domains of manual ability including eating (4 items), dressing (5 items), hygiene and grooming (2 items), play and games (2  items), leisure activities (3 items), and environmental management (1 item). Most of the items (*n* = 14) are achieved using both hands when performed by typically developing children. Many of the bimanual items (*n* = 9) can be managed in several unimanual steps when using an adaptive strategy. Only 1 item was purely unimanual and 2 other items were either unimanual or bimanual, depending on contextual factors. So, “Brushing or combing one’s hair” is commonly performed using 1 hand when hair is short and straight but may require the other hand for untangling long hair for stabilization to avoid pain and facilitate the disentangling. “Putting on a hat” may also be performed using 1 or 2 hands, depending on the type of hat (e.g., putting on a cap requires typically 1 hand while putting on a woolly hat requires 2 hands). The standard errors associated with each item difficulty range from 2.1% to 2.5% of full scale with a mean of 2.2%. The scale presents a non-significant χ^2^ for item–trait interaction (χ^2^ (df): 53.11 (51); *p*-value: 0.39) indicating that, overall, the 17 items contribute to the definition of a unidimensional manual ability measure. This is confirmed by individual item fit statistics showing that all items fit a unidimensional scale: standardized fit residuals are between –1.07 and 1.55 and all individual item χ^2^ *p*-values are ≥ 0.05 (mean: 0.46).

**Table II:** ABILHAND-Kids Young CP calibration

Item	Difficulty (% of full scale)	SE (% of full scale)	Fit			Hands involvement^b^	Precision of 2 hands	Stabilization of 1hand^c^	Strength of 2 hands^c^
			Residual	Chi-square	Prob. *χ*^2^				
			(z)	(*χ*^2^)	(*p*-value)				
01. Drawing a line with a ruler	64.0	2.4	-0.07	5.96	0.11	2B	X	X	
02. Holding a fan of 4-5 playing cards with both hands	63.0	2.3	0.03	1.04	0.79	2C	X	XX	
03. Zipping up a jacket or a coat (attaching both halves of the zipper)^a^	61.9	2.2	0.08	2.82	0.42	2B	X	X	X
04. Opening a bag of chips^a^	61.0	2.3	-0.31	3.53	0.32	2C	X		X
05. Opening a lunch or Tupperware box^a^	53.8	2.2	0.30	0.53	0.91	2B		X	X
06. Turning a key in a door lock	53.1	2.2	0.71	5.76	0.12	1			
07. Taking off a T-shirt^a^	52.9	2.1	0.51	0.98	0.81	2A			X
08. Unwrapping a chocolate bar^a^	51.8	2.1	-0.36	2.76	0.43	2A	X	X	
09. Towelling off after a bath or a shower	50.8	2.1	-0.77	3.78	0.29	2A			X
10. Dealing playing cards one at a time	50.1	2.3	0.15	0.96	0.81	2A		X	
11. Brushing or combing one’s hair	47.6	2.1	-0.27	3.54	0.32	1/2A		(X)	(X)
12. Moving a zipper up and down	47.0	2.1	-0.07	0.83	0.84	2A		X	
13. Drinking from a bowl	43.2	2.1	-0.35	2.15	0.54	2A		XX	X
14. Turning the pages of a comic book (one at a time)	42.0	2.2	-1.07	2.07	0.56	2A		X	
15. Colouring in a drawing with a pencil (colouring outside the drawing outline is allowed)	41.1	2.2	1.55	3.39	0.34	2A		X	
16. Removing an unzipped jacket or coat	38.6	2.2	-0.28	7.48	0.06	2A			(X)
17. Putting on a hat^a^	31.1	2.5	-0.51	5.55	0.14	1/2A			

Items are sorted by decreasing difficulty from top to bottom. Higher logit and % (of full scale) values indicate more difficult activities. SE: standard error; Prob. *χ*^2^: probability of the chi-square.

a activities also included in ABILHAND-Kids (8).

b The most frequently reported experts’ opinion is presented. 1 indicates unimanual activities; 2 indicates bimanual activities manageable in several unimanual steps (2A), requiring stabilization with 1 hand and digital activity with the other (2B), requiring digital activity from both hands (2C).

c XX indicates a stabilization of both hands and (X) indicates that it depends on the contextual factors of the activities (e.g., “Removing an unzipped jacket or coat” may require more or less strength depending on the child’s age and the jacket or coat weight; “Brushing or combing one’s hair” may require more or less precision and strength depending on the hair length and hair entangling).

### Item-patient targeting and internal consistency reliability

**Fig. 1:**
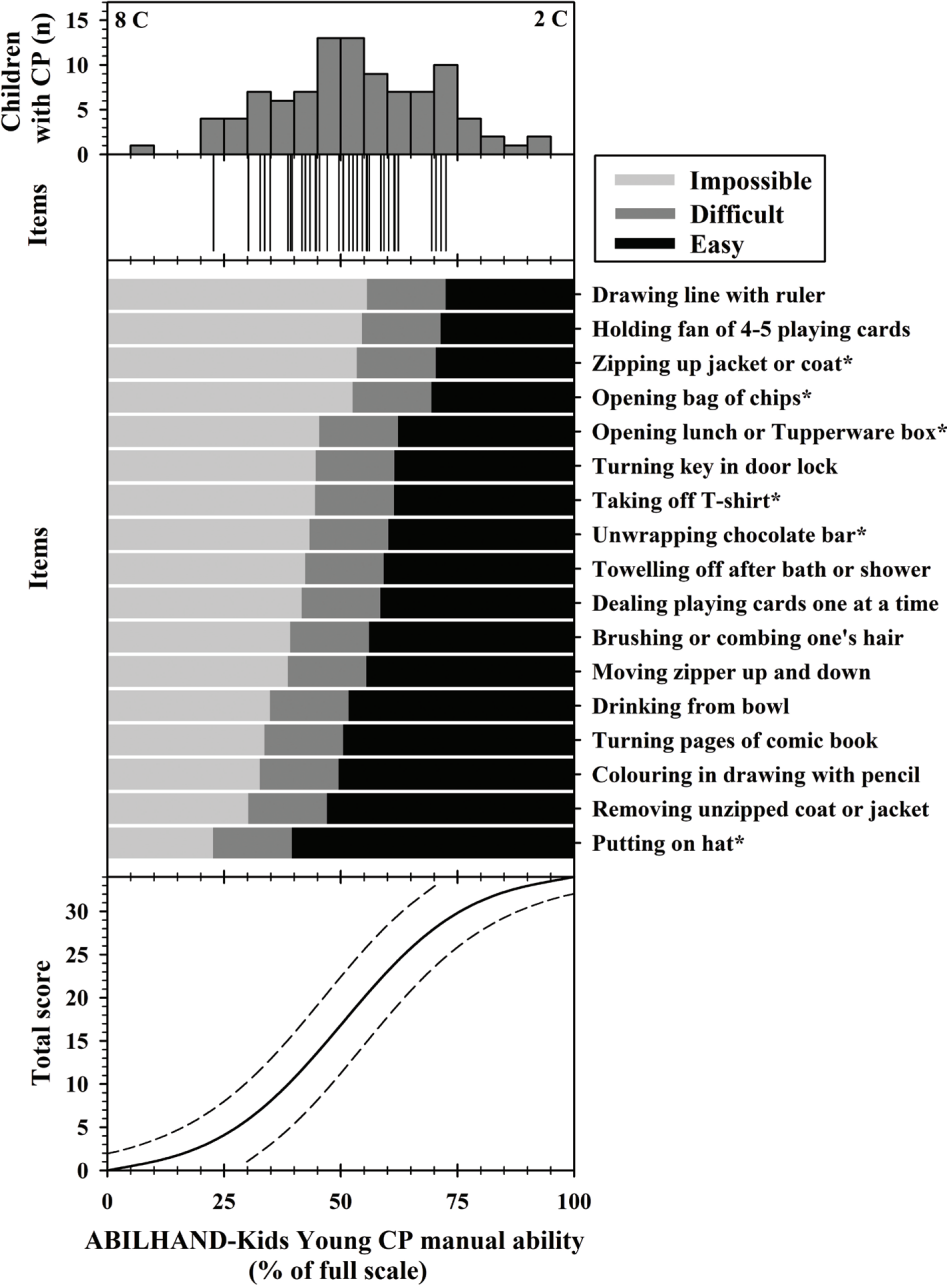
Item map describing the ABILHAND-Kids Young CP scale. Top panel: distribution of ABILHAND-Kids Young CP manual ability measures (expressed as percentage of full scale) of children with cerebral palsy (*n* = 107) according to their caregiver’s perception. Ten children with extreme scores could not be measured by the scale because all activities were either impossible (8C) or easy (2C). Upper middle panel: distribution of the 34 ABILHAND-Kids Young CP item thresholds (17 * 2 thresholds) according to manual ability measure (expressed as percentage of full scale). Lower middle panel: child’s most probable score (“impossible” in light grey, “difficult” in dark grey, and “easy” in black) to each item as a function of the underlying manual ability measure (expressed as percentage of full scale); the thresholds between successive responses to each item are located at –8.4 and +8.4% relative to the item difficulty. The items are sorted, from top to bottom, in order of decreasing difficulty. Items also included in the ABILHAND-Kids questionnaire (8) are highlighted by an asterisk. A manual ability measure of 50% of full scale is by convention set at the average item difficulty. Bottom panel: sigmoidal (S-shaped) curve showing the relationship between total raw scores and ABILHAND-Kids Young CP manual ability measures expressed as percentage (of full scale) units (solid line) and 95% confidence interval, considering the standard errors (dotted lines).

The top and upper middle panels of Fig. 1 show the item-patient targeting. The mean item difficulty is equal to 50% (SD: 9.2) of full scale and the mean children’s manual ability measure is equal to 50.1% (SD: 22.3) indicating that the scale difficulty is extremely well adapted to our sample. Eight children’s caregivers reported a minimum total score (0% of full scale) and 2 reported a maximal total score (100% of full scale) on the questionnaire. The manual ability of their children cannot be measured by ABILHAND-Kids Young CP because all activities are either impossible or easy. However, no substantial floor and ceiling effects were observed on the scale (percentage of children with extreme scores < 15%). The standard errors associated with each child’s measure range from 10.2% to 28.2% of full scale for non-extreme persons with a mean of 13.2%. This mean rises to 15.2% when extreme persons are included as their measures present a standard error of 34.3%.

The person separation index was equal to 0.94, indicating that the scale has high internal consistency, enabling between 5 and 6 manual ability levels to be statistically distinguished in our sample.

### Description of ABILHAND-Kids Young CP

The definition and use of ABILHAND-Kids Young CP are depicted in Fig. 1. According to the distribution of subjects’ measures, 41% of the children in our sample should be able to successfully perform all the listed activities easily or with some difficulty (top and lower middle panels of Fig. 1).

The bottom panel illustrates the sigmoidal relationship between the total raw scores and the manual ability measures (expressed as percentage of full scale and logit units). This relationship is approximately linear between total scores of 9 and 26. Outside of this central range, however, a unitary progression in total score accounts for an increasing amount of manual ability measure. In the central range, the change in manual ability measure corresponding to a unitary increment in the total score from 17 to 18 is equal to 1.6% of full scale. Outside of this central range, it increases up to 9.7% for the same increment in the total score from 0 to 1. This six-fold difference denotes the non-linearity of the total scores and the need to transform the raw scores into linear measures. The conversion between raw total scores and linear measures of ABILHAND-Kids Young CP is given in Table III. Note that the conversion table is only useful without missing data.

**Table III:** Conversion between raw total scores and linear measures of ABILHAND-Kids Young CP

Raw total scores (0–17)	Measures (% of full scale)	SE(% of full scale)	Raw total scores (18–34)	Measures (% of full scale)	SE(% of full scale)
0	0.0	14.6	18	51.8	4.4
1	9.7	10.3	19	53.4	4.4
2	16.4	8.2	20	54.9	4.4
3	21.1	7.1	21	56.6	4.5
4	24.7	6.4	22	58.2	4.5
5	27.7	5.9	23	59.9	4.6
6	30.4	5.5	24	61.7	4.7
7	32.7	5.2	25	63.6	4.8
8	34.9	5.0	26	65.5	5.0
9	36.9	4.9	27	67.7	5.2
10	38.7	4.7	28	70.0	5.5
11	40.5	4.6	29	72.6	5.8
12	42.3	4.5	30	75.6	6.3
13	43.9	4.5	31	79.1	7.0
14	45.5	4.4	32	83.7	8.1
15	47.1	4.4	33	90.4	10.3
16	48.7	4.4	34	100.0	14.5
17	50.3	4.4			

SE: standard error.

The lower middle panel shows the most probable response to a given item as a function of the underlying manual ability measure. For all items, the distance between the 2 thresholds was 16.9% of full scale as the rating scale model was used. By comparing the ability of a given child to the difficulty of each item, it is possible to determine the child’s most probable scores for the items. So, a child with a total raw score of 17 will have a manual ability close to 50% of full scale and would be expected to perform the 3 easiest activities without difficulty; the 10 next intermediate activities with some difficulties; and the 4 most difficult activities would be impossible to perform.

### Scale validity

The opinions of the 10 experts concerning the hand involvement in each activity were consistent with the item difficulty hierarchy. Activities requiring more bimanual involvement tend to be more difficult. Indeed, the most difficult manual activities were those that require digital activity from both hands or stabilization with 1 hand and digital activity with the other. Bimanual activities that can be managed in several unimanual steps when using an adaptive strategy and unimanual activities were the easiest. More specifically, tasks requiring precision from both hands and stabilization from at least 1 hand were the most difficult, followed by tasks requiring strength from both hands. Activities requiring only stabilization from 1 hand (without precision or strength from both hands) were less difficult. Tasks requiring neither precision nor strength from both hands, nor stabilization from at least 1 hand were the easiest.

As expected, no significant effects of gender (*p* = 0.316) and handedness (*p* = 0.369) were observed in the ABILHAND-Kids Young CP measures. On the contrary, children with CP attending regular education (median [Q1–Q3]: 58.7% [46.6–70] of full scale) had significantly (*p* < 0.001) higher manual ability measures than children attending special education (median [Q1–Q3]: 37% [32.6–49.9]) or not schooled (median [Q1–Q3]: 25.5% [0–29.6]). A significant but weak relationship (*r* = 0.22; *p* = 0.023) was observed between manual ability measures and age. As expected, children with diplegia (mean [SD]: 66.7% [20.6] of full scale) or hemiplegia (mean [SD]: 54.8% [18.8]) presented significantly (*p* < 0.001) higher manual abilities than children with quadriplegia (mean [SD]: 35% [22.9]). Finally, significant moderate correlations were also found between ABILHAND-Kids Young CP measures and, respectively, MACS levels (*r* = –0.63; *p* < 0.001) and the PEDI self-care section (*r* = 0.74; *p* < 0.001).

## DISCUSSION

This study allowed the development of ABILHAND-Kids Young CP measuring manual ability in CP children aged from 2 to 7 years old. Seventeen items with robust psychometric qualities were selected following successive Rasch analyses. They were relevant for young children’s daily life, had well-discriminated response categories, shared a similar rating scale, defined a unidimensional scale, were locally independent, and showed an invariant item difficulty hierarchy across various demographic and clinical characteristics. It was particularly important to verify the scale invariance according to the CP clinical form as previous studies (23, 24) showed that item difficulty hierarchy may vary according to the symmetric/asymmetric nature of the disorder. The scale was well targeted on the sample and presented high measurement precision (person separation index = 0.94). Content and construct validities of the scale were also demonstrated.

ABILHAND-Kids Young CP items cover various domains meeting the rehabilitation priorities reported by the parents of young children with CP. Beside mobility, which is also considered by parents as a key issue, dressing, eating, leisure/play, and, to a lesser extent, toileting are reported as the most frequent priorities in 2–5-year-old children with CP (25). Overall, the scale content is in line with parents’ priorities as it mainly includes dressing, eating, and leisure/play activities (29.5%, 23.5%, and 29.5% of the items, respectively). Toileting activities are lacking in the questionnaire. Only 1 item related to toileting was present in the experimental version of the questionnaire, but it was removed as its difficulty changed according to age, CP clinical form, and MACS levels.

The difficulty hierarchy of ABILHAND-Kids Young CP items is congruent with the experts’ clinical opinion concerning the nature of items. Similarly to a previous study in school-aged children (8), bimanual tasks necessarily requiring both hands were more difficult than unimanual activities or activities manageable in several unimanual steps. Tasks demanding bimanual precision (“Holding a fan of 4–5 playing cards with both hands”) were the most difficult items, followed by activities requiring bimanual strength (“Opening a lunch or Tupperware box”). Tasks requiring only stabilization from 1 hand without any bimanual precision or strength (“Turning the pages of a comic book”) were less difficult as the child may potentially use compensatory strategies by stabilizing the objects in other ways (using another body structure or the environment) (2, 8). Activities needing neither bimanual precision nor bimanual strength nor stabilization from 1 hand (“Putting on a hat”) were the easiest. As already previously observed (26), the unimanual item “Turning a key in a door lock” was relatively difficult for our sample. This item requires visuospatial abilities that are impaired in most CP children, even to a minor degree (27). We also hypothesize that for safety reasons (to avoid locking someone in or trapping one’s fingers in the door), few opportunities to try this task are given by young children’s parents. Additionally, this activity might be difficult as it requires broad proximal movements due to the small children’s height. Overall, similar item difficulty hierarchies were found in previous calibrations made in CP children (8, 26). Nevertheless, “Unwrapping a chocolate bar” seems more difficult in our study including pre-school children than in school-aged children (8). We can hypothesize that younger children have not yet developed adaptive strategies enabling them to perform this bimanual activity manageably in several unimanual steps.

Although the ABILHAND-Kids Young CP measurement range is narrow as compared with previous scales (8, 26), it presents a high degree of precision (person separation index = 0.94). The narrow measurement range could be due to the greater homogeneity observed in the manual ability of young children, whose development is still at an early stage. As observed in longitudinal studies (28–30), younger CP children show fewer variations in their self-care scores than older children. Although their grip patterns and quality of movements do not easily improve over the years, manual ability levels may become more heterogeneous with age as children may progressively learn more effective movement strategies depending on their functional and intellectual levels (31). However, the precision of a scale is not only determined by the measurement range, but also by the targeting between the items and subjects’ measures, which was very good in our study. Considering its high internal consistency, ABILHAND-Kids Young CP has the potential to measure a wide range of manual ability levels, giving clinicians the opportunity to follow the evolution of young children with CP and to document changes related to neurorehabilitation. However, the test–retest reliability and responsiveness still need to be investigated.

The cumulative floor/ceiling effects observed in ABILHAND-Kids Young CP (9%) were not substantial. According to their parents, only 2% of children in our sample were able to perform all items easily. All these children were diplegic and able to handle objects easily and successfully (MACS: Level I). In addition, 7% of our sample were unable to perform even 1 ABILHAND-Kids Young CP activity. Most of these children were tetraplegic (6 out of the 8 children; 2 hemiplegics) and all of them handled only a limited selection of easily managed objects (MACS: Level IV) or were unable to handle objects (MACS: Level V). In our study, all children with MACS Level V had a minimum score, suggesting that the questionnaire is not sensitive for children with very severe manipulation problems. Adding very easy items such as “Holding a comfort blanket” or “Throwing a die” could extend its measurement range to lower levels of manual ability.

The relationships between ABILHAND-Kids Young CP measures and demographic/clinical parameters appear not only as a form of scale construct validation but also as a point of clinical interest. The lack of relationships with gender and handedness had already been found in previous studies (8, 24, 32). Although the correlation between age and manual ability was significant, the relationship was weak. This suggests that the questionnaire is more sensitive to the pathologic disruption of manual ability rather than to a maturation of manual ability (8) and that the manual activities accomplishment is more dependent on the CP severity than on age (7). An age relationship was not previously found in young children (24, 32–35) though it seems to exist in little- and non-disabled children (34). ADLs develop gradually during early childhood and are fully mastered around 7–8 years old in typically developing children (19, 20). ABILHAND-Kids Young CP normative values are therefore required to appraise the functional delay of children with CP and to distinguish therapy-related manual ability changes from natural age-related changes. Young children with CP attending regular education had significantly higher manual ability than children attending special education or not schooled. School education may appear as an overall measure of the child’s functioning (36) where more disabled children are generally attending special schools, which are prone to better cope with treatment requirements. As previously shown (2, 35, 37), quadriplegics present more severe hand functioning problems than diplegics or hemiplegics.

Significant moderate correlations were also found between the scale and, respectively, MACS levels and PEDI self-care section. This highlights that our scale is measuring a concept close but not identical to those caught by the MACS and assessed by the PEDI. While the MACS, being a classification tool, is not designed to measure clinical changes after interventions, ABILHAND-Kids Young CP offers higher precision, allowing, for instance, children with MACS Level I to be discriminated across a wide range of manual ability levels (from 9.7 to 100% of full scale). The moderate correlation observed with the PEDI, which is more focused on what the child can do in his/her daily environment, further supports the difference between performance and capability, 2 related but separate concepts (38).

Several limitations are present in the study. Results may be open to selection bias as a convenience sample was used. Moreover, our sample was underrepresented in the MACS Level V (5%) and in the 2-year-old age group (6%). The scale usability for 2-year-olds should therefore be further investigated. However, results are encouraging as the scale enables the measurement of a large range of manual ability levels (from 0.5% to 54.9% of full scale) for these very young children and 83% of them had no missing values or only one. The limited sample size may have resulted in insufficient statistical power to detect potential misfit and larger samples will, therefore, be needed to confirm the unidimensionality of ABILHAND-Kids Young CP. The Mini-MACS (39), more adapted for young children, was not used as it was unavailable at the time of the study. This has potentially introduced a bias, even though a good relationship was observed between MACS and ABILHAND-Kids Young CP. Finally, we applied an absolute critical value of 0.3 to assess local item dependence although more conservative values could also be considered (40). This cut-off was selected because it effectively identified locally dependent items linked by their clinical content and/or underlying manual skills. Nevertheless, the presence of a minor remaining response dependence cannot be excluded, and this may have slightly inflated the estimated scale reliability.

Despite these limitations, ABILHAND-Kids Young CP demonstrates clinical relevance and good psychometric qualities, offering new opportunities to assess children’s manual ability from the earliest age of 2, to monitor manual ability changes over time by bridging the gap with ABILHAND-Kids, and to potentially assess neurorehabilitation outcomes. Moreover, the scale is available on https://www.rehab-scales.org and an online analysis is free-to-use for clinical and research applications. The validation of an instrument is an ongoing process, and not all psychometric properties of a scale can be established in a single study. Therefore, it will be important in the future to study the test–retest reliability and the responsiveness of ABILHAND-Kids Young CP to ensure its ability to detect clinical changes in the manual ability of young children with CP.

## Supplementary Material


