# Supplementary material for: ABILHAND-KIDS YOUNG CP: A MEASURE OF MANUAL ABILITY IN YOUNG CHILDREN WITH CEREBRAL PALSY AGED 2 TO 7
Source: J Rehabil Med. 2025 Aug 20;57:42691. doi: 10.2340/jrm.v57.42691 (PMC12379724; doi:10.2340/jrm.v57.42691)
Supplement: Supplementary file 1 [file JRM-57-42691-s1.pdf]

Appendix S1

Critical review of upper limb performance measures in young children with CP

Classifications as the MACS (1) or the Mini-MACS (2) are useful to classify the severity of disturbance of manual ability but are not designed to measure changes after interventions and should thus not be used as outcome measures (3, 4). Some reported tools, such as the Pediatric Evaluation of Disability Inventory (PEDI) (5), the PEDI-Computer Adaptive Test (PEDI-CAT) (6), and the Assessment of Motor and Process Skills (AMPS) (7), are originally developed as generic measures, which is likely to induce a risk of measurement error since the difficulty of activities is diagnosis-dependent (8). Children's Hand-use Experience Questionnaire (CHEQ) (9) is an interesting tool measuring children's perceived performance and satisfaction when using the affected hand in bimanual activities. Although it provides relevant clinical information, it requires more psychometric evidence (4). The high number of missing scores (when activity is not performed independently or is not achieved with both hands) is another problem (3) preventing from the measurement of children with severe CP. Other instruments, such as Infant and Pediatric Motor Activity Log family tools (IMAL/PMAL) (10-13), Assisting Hand Assessment (AHA) family tools (14-18), Caregiver Functional Use Survey (CFUS) family tools (19, 20), and Parent-rated Hand-Use-at-Home questionnaire (HUH) (21), are also only focused on the assessment of children with unilateral CP.

Only few specific tools allow children with bilateral CP to be measured. The Both Hands Assessment (BoHA) (22), developed for children aged 18 months to 12 years, is a videotaped tool measuring how effectively children with bilateral CP use both hands in bimanual activities. Although BoHA predominantly measures the activity domain of the International Classification of Functioning, Disability and Health (ICF-CY) (23), it captures the mobility subdomain rather than the self-care subdomain (24). Moreover, it is not applicable for children with unilateral CP. The self-care domain of the second version of Child Engagement in Daily Life Measure (CEDL2) (25), developed for children aged 18 months to 12 years, is a parent-report questionnaire measuring the degree the child participates in his/her self-care activities (i.e., child's level of independence and need for assistance to perform self-care activities). Although this second version addresses the limitations of the original tool (26), it requires further investigations about their psychometric qualities as some items present disordered response scale and many items show a differential item functioning according to the Gross Motor Function Classification levels. Children's Arm

Rehabilitation Measure (CHARM) (27), developed for children aged 5 to 16 years, is a parent-report questionnaire measuring the child's capacity to achieve activities requiring upper limb use. Although interesting as it was based on the most common clinically relevant goals for children with CP (27), CHARM is difficult to use as each item has different responses to read and consider and it requires further psychometric testing (i.e., responsiveness, additional validity evidence, test-retest reliability).

References

1. Eliasson AC, Krumlinde-Sundholm L, Rösblad B, Beckung E, Arner M, Ohrvall AM, et al. The Manual Ability Classification System (MACS) for children with cerebral palsy: scale development and evidence of validity and reliability. Dev Med Child Neurol 2006; 48: 549–554. https://doi.org/10.1017/S0012162206001162
2. Eliasson A-C, Ullenhag A, Wahlström U, Krumlinde-Sundholm L. Mini-MACS: development of the Manual Ability Classification System for children younger than 4 years of age with signs of cerebral palsy. Dev Med Child Neurol 2017; 59: 72–78. https://doi.org/10.1111/dmcn.13162
3. Amer A, Eliasson A-C, Peny-Dahlstrand M, Hermansson L. Validity and test-retest reliability of Children's Hand-use Experience Questionnaire in children with unilateral cerebral palsy. Dev Med Child Neurol 2016; 58: 743–749. https://doi.org/10.1111/dmcn.12991
4. Wallen M, Stewart K. Upper limb function in everyday life of children with cerebral palsy: description and review of parent report measures. Disabil Rehabil 2015; 37: 1353–1361. https://doi.org/10.3109/09638288.2014.963704
5. Haley SM, Coster WJ, Ludlow LH, Haltiwanger JT, Andrellos PJ. Pediatric Evaluation of Disability Inventory (PEDI). Development, standardization, and administration manual, version 1.0. Boston (MA): New England Medical Center Hospitals; 1992.
6. Dumas HM, Fragala-Pinkham MA, Haley SM, Ni P, Coster W, Kramer JM, et al. Computer adaptive test performance in children with and without disabilities: Prospective field study of the PEDI-CAT. Disabil Rehabil 2012; 34: 393–401. https://doi.org/10.3109/09638288.2011.607217

7. Fisher AG, Bray Jones K. Assessment of Motor and Process Skills. Vol. 1: Development, standardization, and administration manual (7th Rev. ed.). Fort Collins: Three Star Press; 2012.
8. Arnould C, Vandervelde L, Batcho CS, Penta M, Thonnard J-L. Can manual ability be measured with a generic ABILHAND scale? A cross-sectional study conducted on six diagnostic groups. BMJ Open 2012; 2: e001807. https://doi.org/10.1136/bmjopen-2012-001807
9. Sköld A, Hermansson LN, Krumlinde-Sundholm L, Eliasson A-C. Development and evidence of validity for the Children's Hand-use Experience Questionnaire (CHEQ). Dev Med Child Neurol 2011; 53: 436–442. https://doi.org/10.1111/j.1469-8749.2010.03896.x
10. Lowes LP, Mayhan M, Orr T, Batterson N, Tonneman JA, Meyer A, et al. Pilot study of the efficacy of constraint-induced movement therapy for infants and toddlers with cerebral palsy. Phys Occup Ther Pediatr 2014; 34: 4–21. https://doi.org/10.3109/01942638.2013.810186
11. Taub E, Ramey SL, DeLuca S, Echols K. Efficacy of constraint-induced movement therapy for children with cerebral palsy with asymmetric motor impairment. Pediatrics 2004; 113: 305–312. https://doi.org/10.1542/peds.113.2.305
12. Uswatte G, Taub E, Griffin A, Vogtle L, Rowe J, Barman J. The pediatric motor activity log-revised: assessing real-world arm use in children with cerebral palsy. Rehabil Psychol 2012; 57: 149–158. https://doi.org/10.1037/a0028516
13. Wallen M, Bundy A, Pont K, Ziviani J. Psychometric properties of the Pediatric Motor Activity Log used for children with cerebral palsy. Dev Med Child Neurol 2009; 51: 200–208. https://doi.org/10.1111/j.1469-8749.2008.03157.x
14. Greaves S, Imms C, Dodd K, Krumlinde-Sundholm L. Development of the Mini-Assisting Hand Assessment: evidence for content and internal scale validity. Dev Med Child Neurol 2013; 55: 1030–1037. https://doi.org/10.1111/dmcn.12212
15. Holmefur MM, Krumlinde-Sundholm L. Psychometric properties of a revised version of the Assisting Hand Assessment (Kids-AHA 5.0). Dev Med Child Neurol 2016; 58: 618–624. https://doi.org/10.1111/dmcn.12939
16. Krumlinde-Sundholm L, Eliasson AC. Development of the Assisting Hand Assessment: A Rasch-built Measure intended for Children with Unilateral Upper Limb Impairments. Scand J Occup Ther 2003; 10: 16–26. https://doi.org/10.1080/11038120310004529

17. Krumlinde-Sundholm L, Holmefur M, Kottorp A, Eliasson AC. The Assisting Hand Assessment: current evidence of validity, reliability, and responsiveness to change. Dev Med Child Neurol 2007; 49: 259–264. https://doi.org/10.1111/j.1469-8749.2007.00259.x
18. Louwers A, Beelen A, Holmefur M, Krumlinde-Sundholm L. Development of the Assisting Hand Assessment for adolescents (Ad-AHA) and validation of the AHA from 18 months to 18 years. Dev Med Child Neurol 2016; 58: 1303–1309. https://doi.org/10.1111/dmcn.13168
19. Charles JR, Wolf SL, Schneider JA, Gordon AM. Efficacy of a child-friendly form of constraint-induced movement therapy in hemiplegic cerebral palsy: a randomized control trial. Dev Med Child Neurol 2006; 48: 635–642. https://doi.org/10.1017/S0012162206001356
20. Gordon AM, Charles J, Wolf SL. Efficacy of constraint-induced movement therapy on involved upper-extremity use in children with hemiplegic cerebral palsy is not age-dependent. Pediatrics 2006; 117: e363–e373. https://doi.org/10.1542/peds.2005-1009
21. Geerdink Y, Aarts P, van der Holst M, Lindeboom R, Van Der Burg J, Steenbergen B, et al. Development and psychometric properties of the Hand-Use-at-Home questionnaire to assess amount of affected hand-use in children with unilateral paresis. Dev Med Child Neurol 2017; 59: 919–925. https://doi.org/10.1111/dmcn.13449
22. Elvrum A-KG, Zethræus B-M, Vik T, Krumlinde-Sundholm L. Development and Validation of the Both Hands Assessment for Children With Bilateral Cerebral Palsy. Phys Occup Ther Pediatr 2018; 38: 113–126. https://doi.org/10.1080/01942638.2017.1318431
23. World Health Organization. The International Classification of Functioning, Disability and Health – Children & Youth Version – ICF-CY. Geneva: WHO; 2007.
24. Arnould C. Practical Considerations of the Both Hands Assessment (BoHA): A commentary on « Development and Validation of the Both Hands Assessment for Children with Bilateral Cerebral Palsy ». Phys Occup Ther Pediatr 2018; 38: 127–129. https://doi.org/10.1080/01942638.2018.1433428
25. Chiarello LA, Alghamdi MS, McCoy SW, Avery L, Palisano RJ. Child engagement in daily life measure V2: validation of psychometric properties for children with cerebral palsy. Disabil Rehabil 2023; 45: 3912–3921. https://doi.org/10.1080/09638288.2022.2140849
26. Chiarello LA, Palisano RJ, McCoy SW, Bartlett DJ, Wood A, Chang H-J, et al. Child Engagement in Daily Life: a measure of participation for young children with cerebral

palsy. Disabil Rehabil 2014; 36: 1804–1816. https://doi.org/10.3109/09638288.2014.882417

27. Preston N, Horton M, Levesley M, Mon-Williams M, O'Connor RJ. Development of a parent-reported questionnaire evaluating upper limb activity limitation in children with cerebral palsy. Physiother Res Int 2018; 23: e1684. https://doi.org/10.1002/pri.1684

Appendix S2

Item selection criteria

From the 80 items of the experimental questionnaire, successive analyses were used to select items presenting good psychometric qualities to constitute the final ABILHAND-Kids Young CP scale:

1) Relevance: an activity is considered as relevant when it belongs to the daily life of most children with CP. Like previous studies (1, 2), only items with a response rate ≥ 80% were retained, as these activities are usually achieved by the children of our sample.

2) Ordered thresholds of the response categories: it is essential to verify that the three-level scale is used as postulated a priori, namely, the higher the response category, the higher the manual ability level. If the anticipated order of response categories is verified, respondents of children with a higher manual ability must always choose a higher response to any given item than respondents of children with a lower ability, and the children of respondents selecting a higher response for a given item should always demonstrate higher manual ability (3). When these conditions are not met, the order of thresholds between successive response categories are reversed, indicating that the rating scale is not used as anticipated (4). Only items showing ordered thresholds of the response categories were retained.

3) Rating scale model: though all items were answered according to the same 3-level scale, the relative threshold locations (relative to the item location) may vary across items, leading to different measurement ranges for which the middle category (i.e., "difficult") is the most probable. The use of a rating scale model (i.e., a model which forced all items to share the same relative threshold locations) was preferred as it favors an easily clinical interpretation of the scores (5). Therefore, items with relative threshold locations significantly different from the average (Z-test) were removed.

4) Unidimensionality: unidimensionality implies that no attribute of a person besides manual ability is theorized to account for the probability of choosing a given response to a given item, even if the subject is characterized by many other attributes. Only unidimensional items presenting standardized residuals (i.e., differences between observed and expected scores divided by the standard deviation of the expected score for each of the 4-class interval [CI] of increasing ability levels) inside the range ± 2.5 or a p-value of the χ^2 fit statistic (computed over 4 CI of increasing ability levels) ≥ 0.05 were retained (6). Simulation studies

have shown that good fit statistics may be reported when a scale was multidimensional (7). Therefore, a Principal Component Analysis (PCA) was also performed on the residuals (i.e., the differences between observed and expected scores) to identify the factor that best explains data variations not explained by the variable modeled by the Rasch model (i.e., manual ability). Simulation studies (8, 9) suggest that an eigenvalue of the first PCA residual factor below 2 indicates that the potential presence of a secondary dimension is not substantial enough to distort unidimensional measurement. Independent t-tests were also used to compare the estimates for each subject's ability, deriving from the highest positive and negative loadings items (correlated at ≥ 0.3 with the first PCA residual factor) (7, 10). The scale is considered as unidimensional when the percentage of tests outside the range ± 1.96 is less than 5% (7).

5) No differential item functioning (DIF): unidimensionality also implies that children with identical manual ability levels but different demographic or clinical characteristics should have the same probability of succeeding in any particular item (3). If this is not the case, the item presents a "differential functioning". The invariance of the item difficulty hierarchy was tested for age (≤ 4 years old vs. > 4 years old, with 4 years old as the median), gender, clinical form of CP (hemiplegia vs. diplegia vs. quadriplegia), and MACS levels (MACS=I-II vs. MACS ≥ III) (11). DIF was measured by computing a two-way analysis of variance (ANOVA) on the standardized residuals of 4 CIs of increasing ability levels; the first factor was the investigated child's characteristics (age, gender, clinical form of CP, and MACS levels) and the second factor was the CIs (6, 12). Items presenting DIF across age, gender, clinical form of CP, and MACS levels, as highlighted by a significant main effect for the first patient factor, were removed.

6) Local independency: local dependency occurs when the scores attributed to the subjects to an item depend on the scores given to another item of the same scale (13). Local dependency can be identified through the correlation matrix of the residuals. Local dependency is not a threat to the psychometric properties of the scale if the correlations between standardized item residuals remain low (14). Residual correlations between two items higher than 0.3 (indicating potential local dependency) led to the suppression of the item with the worse psychometric qualities.

References

1. Bleyenheuft Y, Paradis J, Renders A, Thonnard J-L, Arnould C. ACTIVLIM-CP a new Rasch-built measure of global activity performance for children with cerebral palsy. Res Dev Disabil 2017; 60: 285–294. https://doi.org/10.1016/j.ridd.2016.10.005
2. Durez P, Fraselle V, Houssiau F, Thonnard J-L, Nielens H, Penta M. Validation of the ABILHAND questionnaire as a measure of manual ability in patients with rheumatoid arthritis. Ann Rheum Dis 2007; 66: 1098–1105. https://doi.org/10.1136/ard.2006.056150
3. Penta M, Arnould C, Decruynaere C. Développer et interpréter une échelle de mesure. Applications du modèle de Rasch. Sprimont: Mardaga; 2005.
4. Bouffioulx E, Arnould C, Thonnard J-L. SATIS-Stroke: A satisfaction measure of activities and participation in the actual environment experienced by patients with chronic stroke. J Rehabil Med 2008; 40: 836–843. https://doi.org/10.2340/16501977-0272
5. Arnould C, Penta M, Renders A, Thonnard J-L. ABILHAND-Kids: a measure of manual ability in children with cerebral palsy. Neurology 2004; 63: 1045–1052. https://doi.org/10.1212/01.wnl.0000138423.77640.37
6. Andrich D, Sheridan B. Interpreting RUMM2020: part II, polytomous data RUMM. Perth: Laboratory Pty Ltd; 2005.
7. Smith EV. Detecting and evaluation the impact of multidimensionality using item fit statistics and principal component analysis of residuals. J Appl Meas 2002; 3: 205–231.
8. Linacre JM, Tennant A. More about critical eigenvalue sizes in standardized-residual principal components analysis (PCA). Rasch Meas Trans 2009; 23: 1228. https://www.rasch.org/rmt/rmt233f.htm
9. Raîche G. Critical Eigenvalue Sizes (Variances) in Standardized Residual Principal Components Analysis (PCA). Rasch Meas Trans 2005, 19: 1012. https://www.rasch.org/rmt/rmt191h.htm
10. Smith EV, Conrad KM, Chang K, Piazza J. An introduction to Rasch measurement for scale development and person assessment. J Nurs Meas 2002; 10: 189–206. https://doi.org/10.1891/jnum.10.3.189.52562
11. Eliasson AC, Krumlinde-Sundholm L, Rösblad B, Beckung E, Arner M, Ohrvall AM, et al. The Manual Ability Classification System (MACS) for children with cerebral palsy: scale development and evidence of validity and reliability. Dev Med Child Neurol 2006; 48: 549–554. https://doi.org/10.1017/S0012162206001162

12. Pallant JF, Tennant A. An introduction to the Rasch measurement model: an example using the Hospital Anxiety and Depression Scale (HADS). Br J Clin Psychol 2007; 46: 1–18. https://doi.org/10.1348/014466506x96931
13. Embretson SE, Reise SP. Item response theory for psychologists. Mahwah (NJ): Lawrence Erlbaum Associates Publishers; 2000.
14. Andrich D, Marais I. A Course in Rasch Measurement Theory: Measuring in the Educational, Social and Health Sciences. Singapore: Springer Nature Singapore; 2019. https://doi.org/10.1007/978-981-13-7496-8
